# Supplementary material for: Data driven mixed effects modeling of the dual process framework of addiction among individuals with alcohol use disorder
Source: PLoS One. 2023 Aug 7;18(8):e0265168. doi: 10.1371/journal.pone.0265168 (PMC10406226; doi:10.1371/journal.pone.0265168)
Supplement: S1 File — (PDF) [file pone.0265168.s001.pdf]

Supporting information

S1 MCMC Chain Convergence

To illustrate MCMC chain convergence to the stationary posterior distributions, we report the Monte Carlo error and Geweke p-values for each of the population level parameters in Table S1. All four parameters pass the convergence test at the $\alpha = 0.05$ confidence level. Convergence statistics for each of the 148 random effect chains display similar results, with a median Geweke p-value of 0.9623. The population level parameter chains are displayed in Fig S1. Autocorrelation statistics are reported in Table S2 to illustrate the mixing speed of the chains. A quick decrease in autocorrelation towards zero (as depicted in Fig S1 for each of the four population parameters) indicates that the correlation between samples is greatly decreased as the spacing of samples (or k lag) increases. A small k lag indicates a fast-mixing chain. Ideally, the total runtime of the MCMC chain should be many times the amount of time that it takes for the autocorrelation to decrease to practically zero [62]. We report both the k lag and the ratio of the MCMC chain length to the k lag—N/k for $N = 500,000$—for each of four threshold autocorrelation values, $\varepsilon = \{0.1, 0.05, 0.01, 0.005\}$, in Table S2 to illustrate that this criteria is met. The largest k lag occurs for parameter d_3, but even with our strictest threshold value of $\varepsilon = 0.005$, our MCMC chain length is still roughly 79 times as long as the lag. The 148 individual level random effects MCMC chains also satisfy our criteria for autocorrelation, with a median N/k value of 106.

**Parameter**	**Mean**	**Std. Dev.**	**MC Error**	**Geweke Value**
a_2	0.9944	0.0015	0.00003	0.99997
a_3	-0.4539	0.0990	0.00089	0.99448
d_2	0.4110	0.0527	0.00051	0.99106
d_3	-0.0561	0.0693	0.00197	0.98994

Table S1. Convergence statistics for population level parameter chains. All four population level chains pass the convergence test at the $\alpha = 0.05$ confidence level.

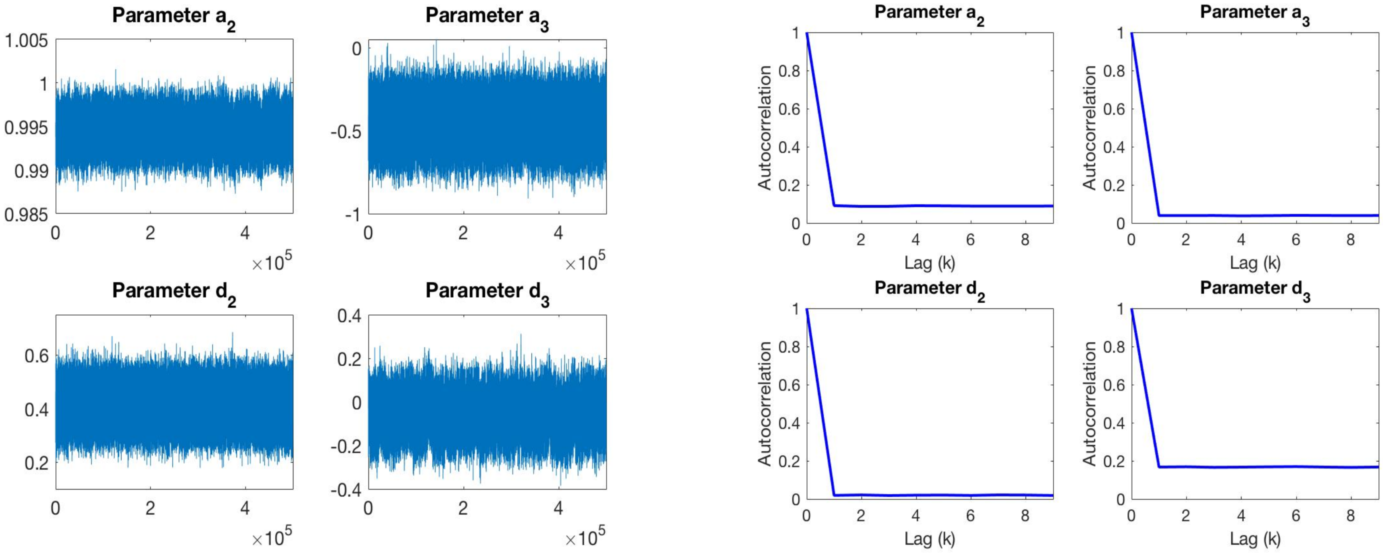

Fig S1. MCMC chains (left) and autocorrelation plots (right) for the four population level parameters. All chains are well-mixed and converge to their stationary posterior distributions, as affirmed by the Geweke statistics reported in Table S1. All four autocorrelation plots decay quickly toward zero within the first couple iterations, indicating a decreased correlation between parameter candidates spaced k samples apart. All autocorrelations continue to gradually decrease toward zero as k increases, as detailed in Table S2.

S2 Sensitivity Analysis Figures

Figs S2 - S7.

	$\varepsilon = 0.1$		$\varepsilon = 0.05$		$\varepsilon = 0.01$		$\varepsilon = 0.005$	
**Parameter**	k	N/k	k	N/k	k	N/k	k	N/k
a_2	1	500000	665	751.88	2487	201.05	2987	167.392
a_3	1	500000	1	500000	1397	357.91	1752	285.39
d_2	1	500000	1	500000	535	934.58	969	516.00
d_3	653	765.70	1698	294.46	5134	97.39	6308	79.26

Table S2. Autocorrelation statistics. For each of the four population level parameters, k lags for four different thresholds are provided, along with the N/k where $N = 500000$, the utilized sample size for the MCMC procedure.

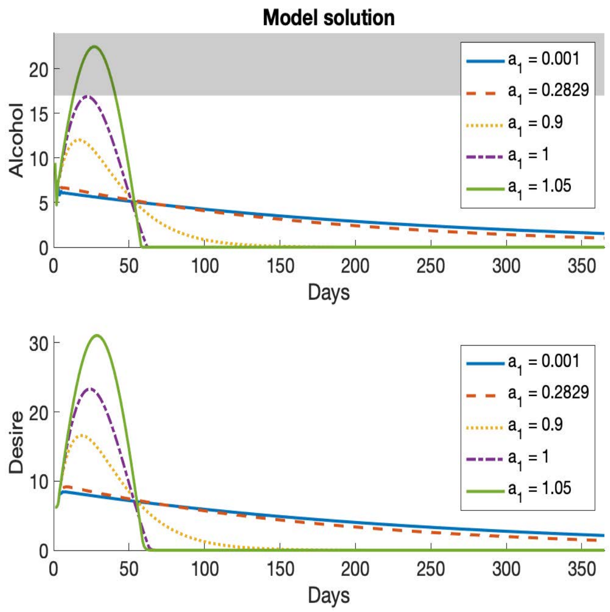
Model solution
Alcohol
Desire
Days
a_1 = 0.001
a_1 = 0.2829
a_1 = 0.9
a_1 = 1
a_1 = 1.05

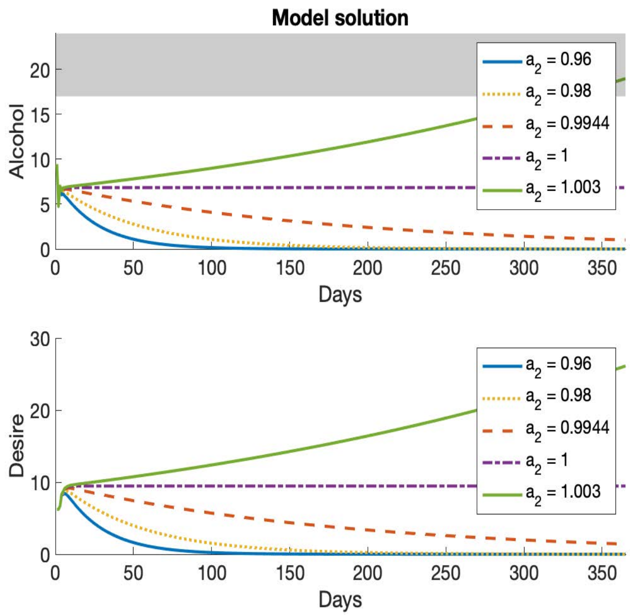
Model solution
Alcohol
Desire
Days
a_2 = 0.96
a_2 = 0.98
a_2 = 0.9944
a_2 = 1
a_2 = 1.003

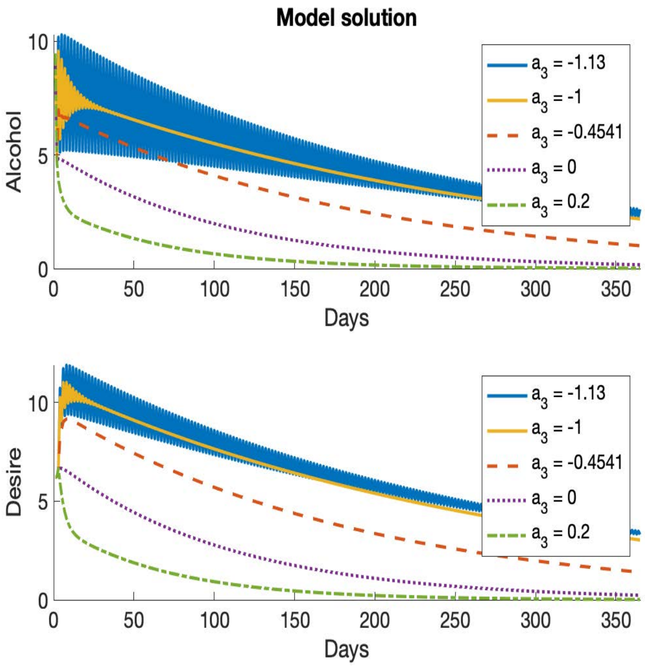
Model solution
Alcohol
Desire
Days
a_3 = -1.13
a_3 = -1
a_3 = -0.4541
a_3 = 0
a_3 = 0.2

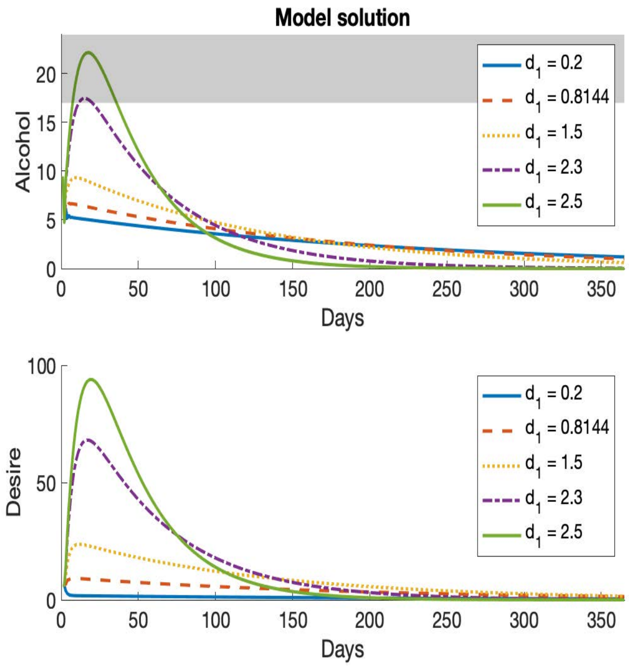
Model solution
Alcohol
Desire
Days
d_1 = 0.2
d_1 = 0.8144
d_1 = 1.5
d_1 = 2.3
d_1 = 2.5

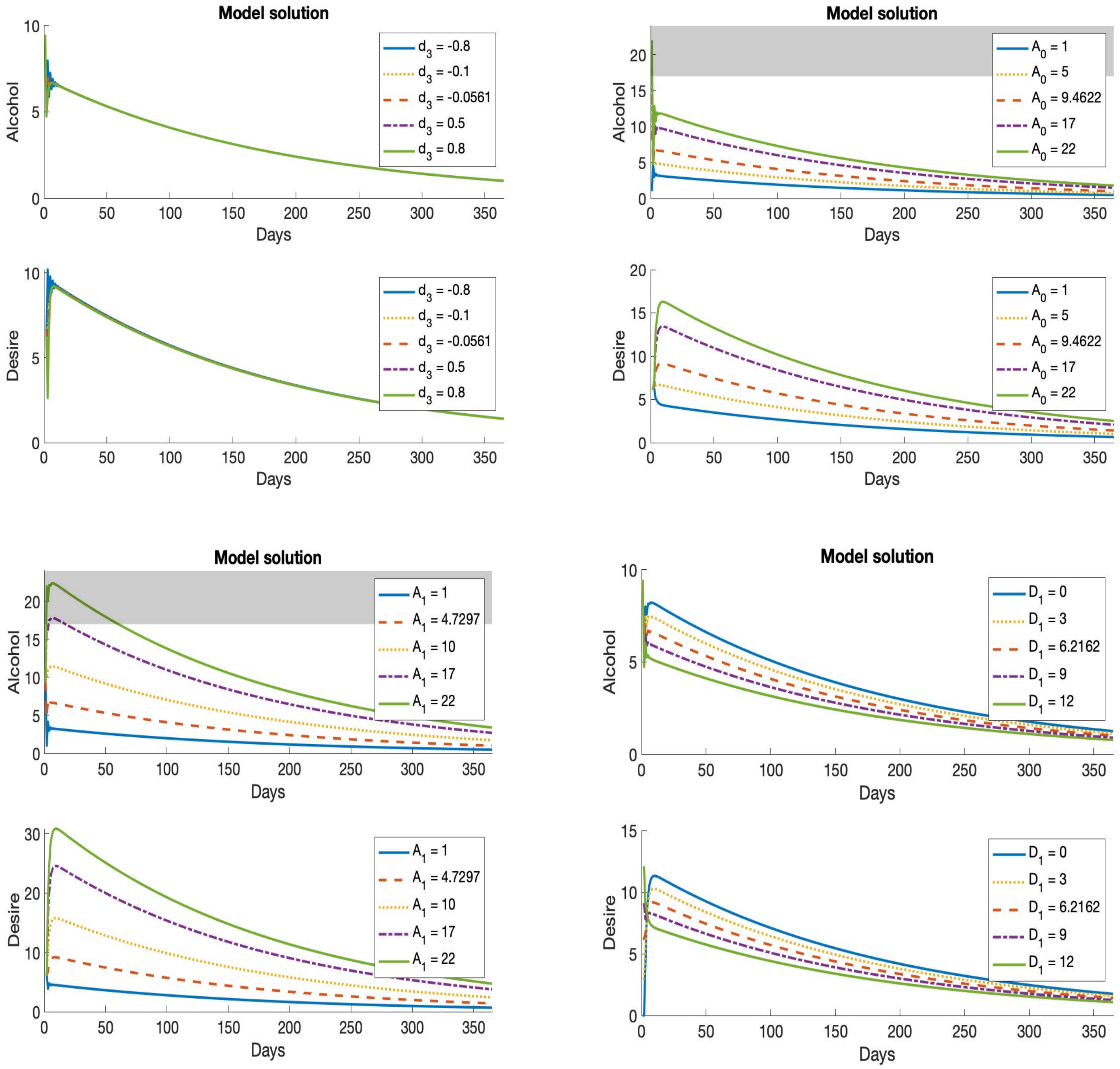

Fig S2. One-at-a-time sensitivity analyses for set $\{a_1, a_2, a_3, d_1, d_3, A_0, A_1, D_1\}$. Note that corresponding simulations for d_2 are shown in Fig 4. For each plot, all other parameters are fixed at their mean values from Table 3 across all simulations. The gray bar represents threshold at which outliers were flattened, indicating a dangerous level of drinking.

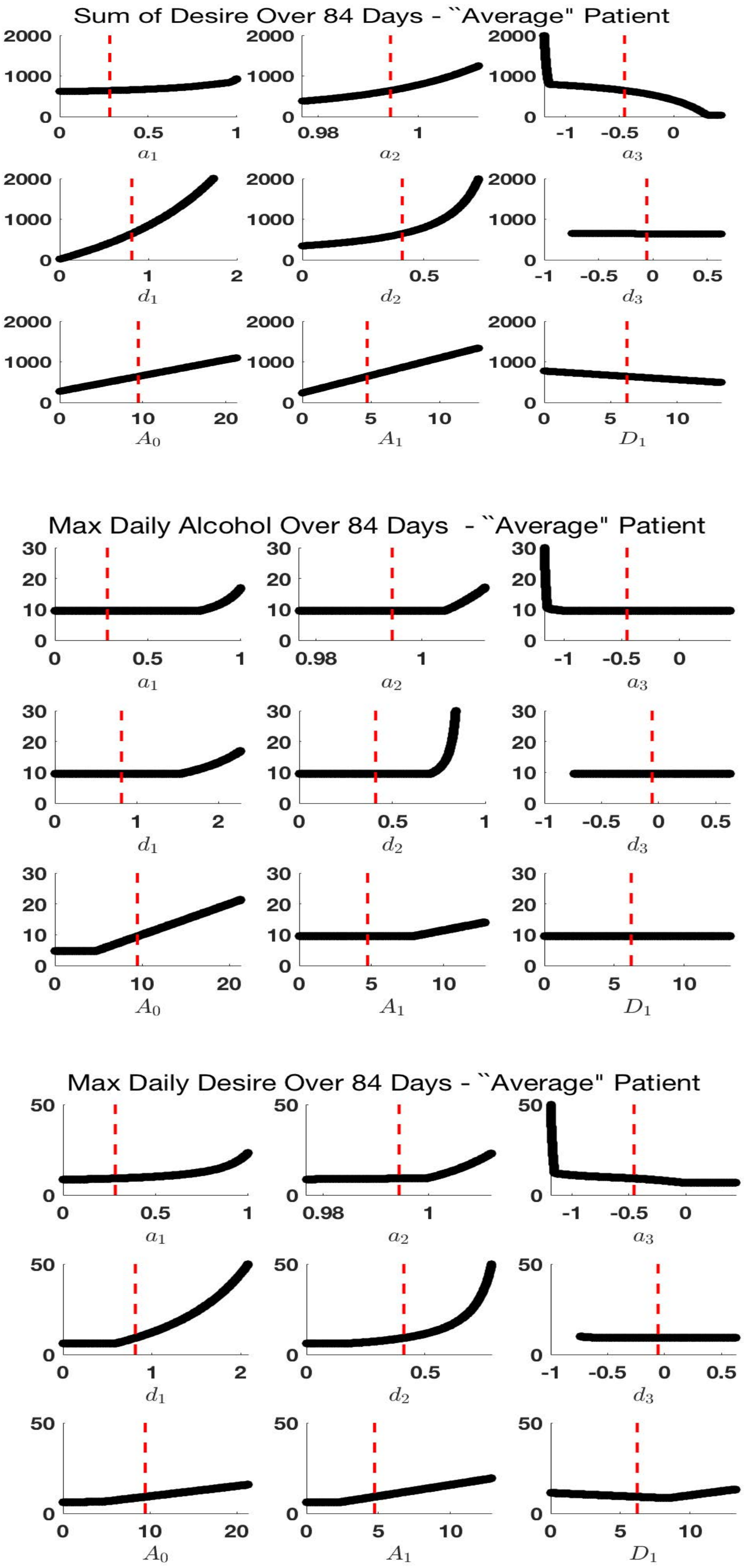

Fig S3. Relationships between varying single parameter values in the Latin Hypercube Sampling and the remaining three output measures for 84 day observation period using the population level parameter set. The majority of plots appear to be monotonic, suggesting the PRCC is an appropriate measure to consider.

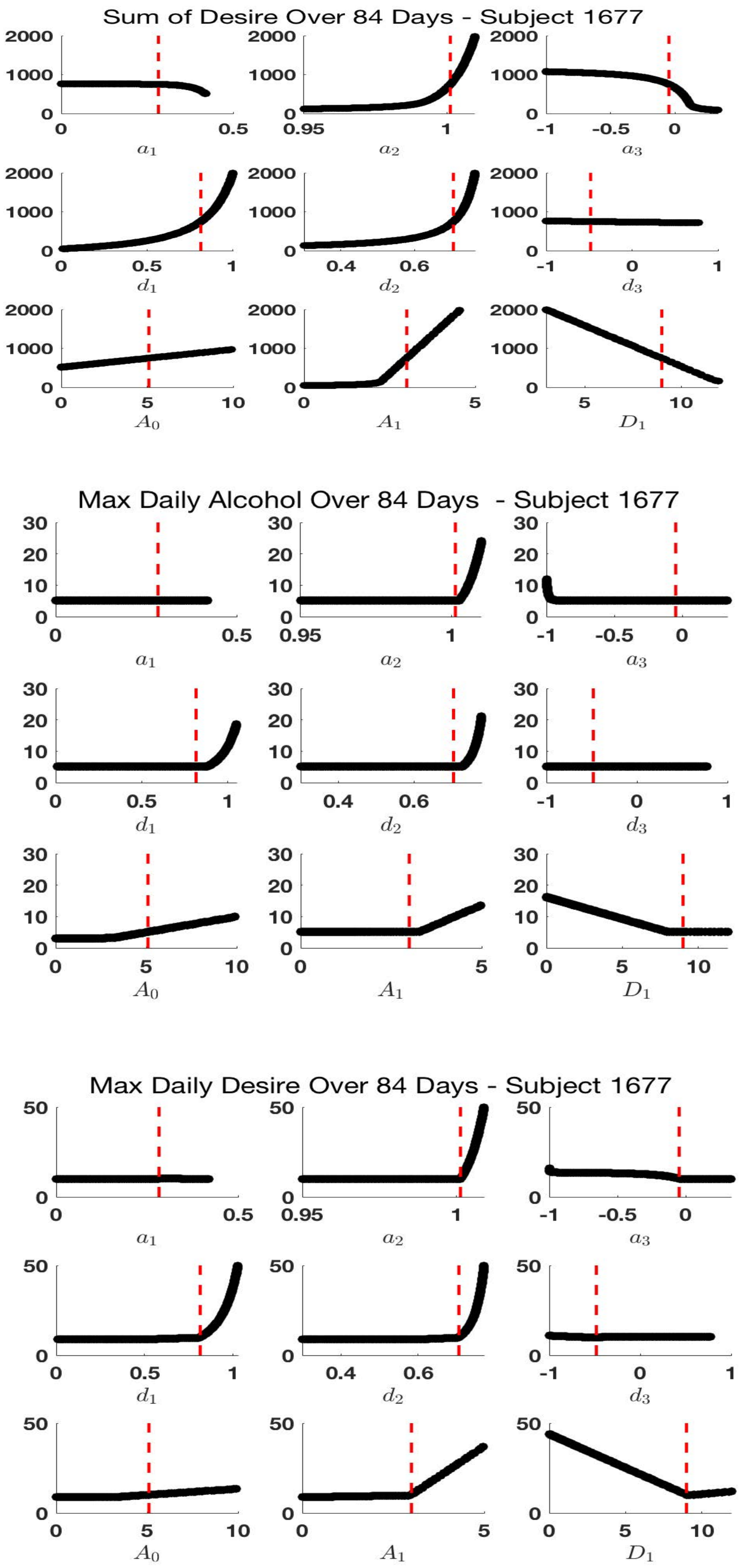

Fig S4. Relationships between varying single parameter values in the Latin Hypercube Sampling and the remaining three output measures for 84 day observation period using parameter set for Subject 1677. The majority of plots appear to be monotonic, suggesting the PRCC is an appropriate measure to consider.

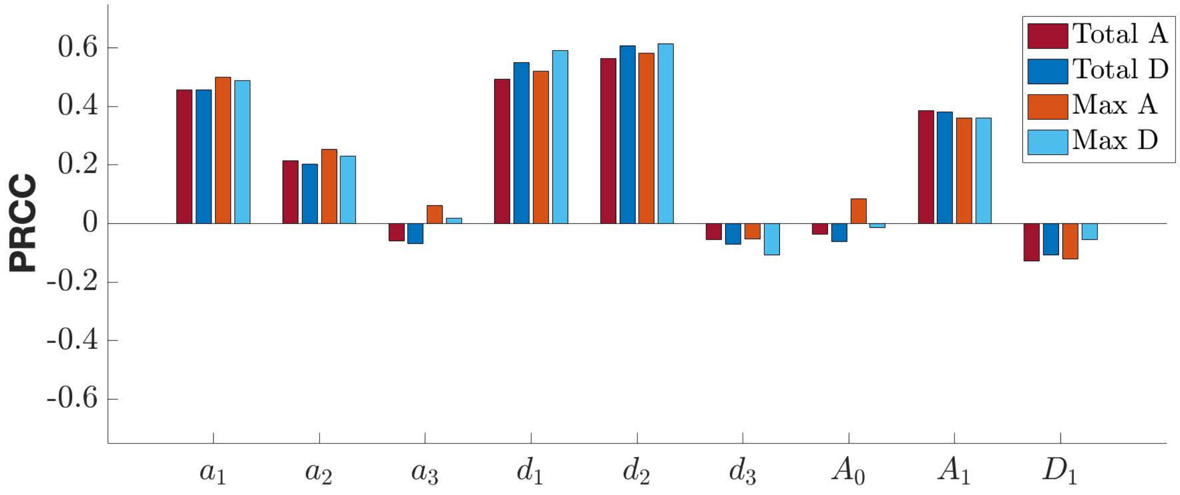

Fig S5. Sensitivity analysis for model parameter values. Partial Rank Correlation Coefficient (PRCC) for each parameter in the Latin Hypercube Sampling for the the output measures after running the model for one year.

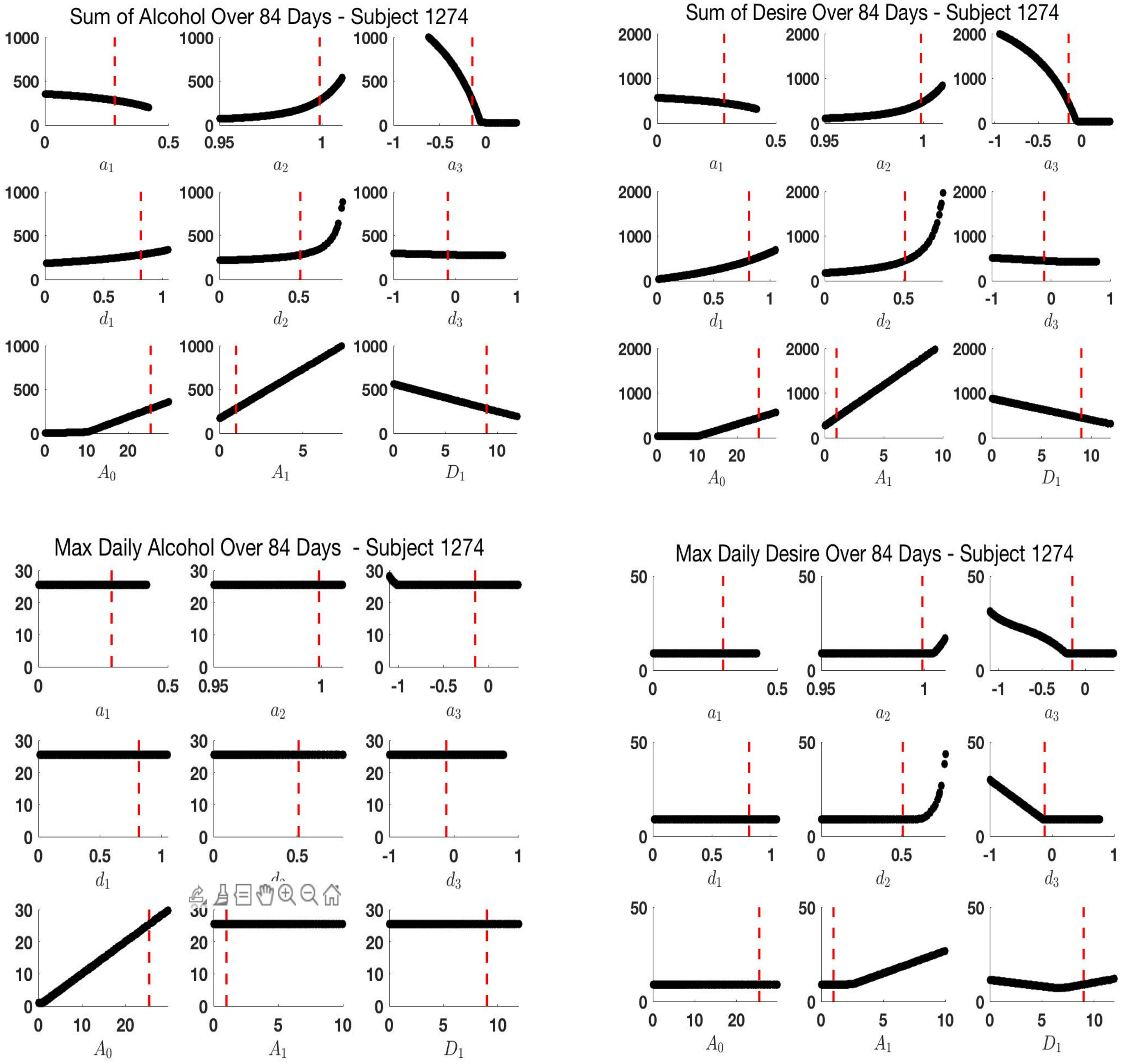

Fig S6. Relationships between varying single parameter values in the Latin Hypercube Sampling and all four output measures for 84 day observation period using parameter set for Subject 1274. The majority of plots appear to be monotonic, suggesting the PRCC is an appropriate measure to consider. We note that a_2 and a_3 appear to be the parameters with the greatest importance. It is apparent, however, particularly when also considering Fig 2, that Subject 1274's behavior reaches what might be considered a steady state. He drinks consistently at a high-risk level with a steady level of desire. For this person, mechanisms to target for effective intervention have yet to be identified. It may be that top-down stimuli will be particularly important mechanisms for this individual.

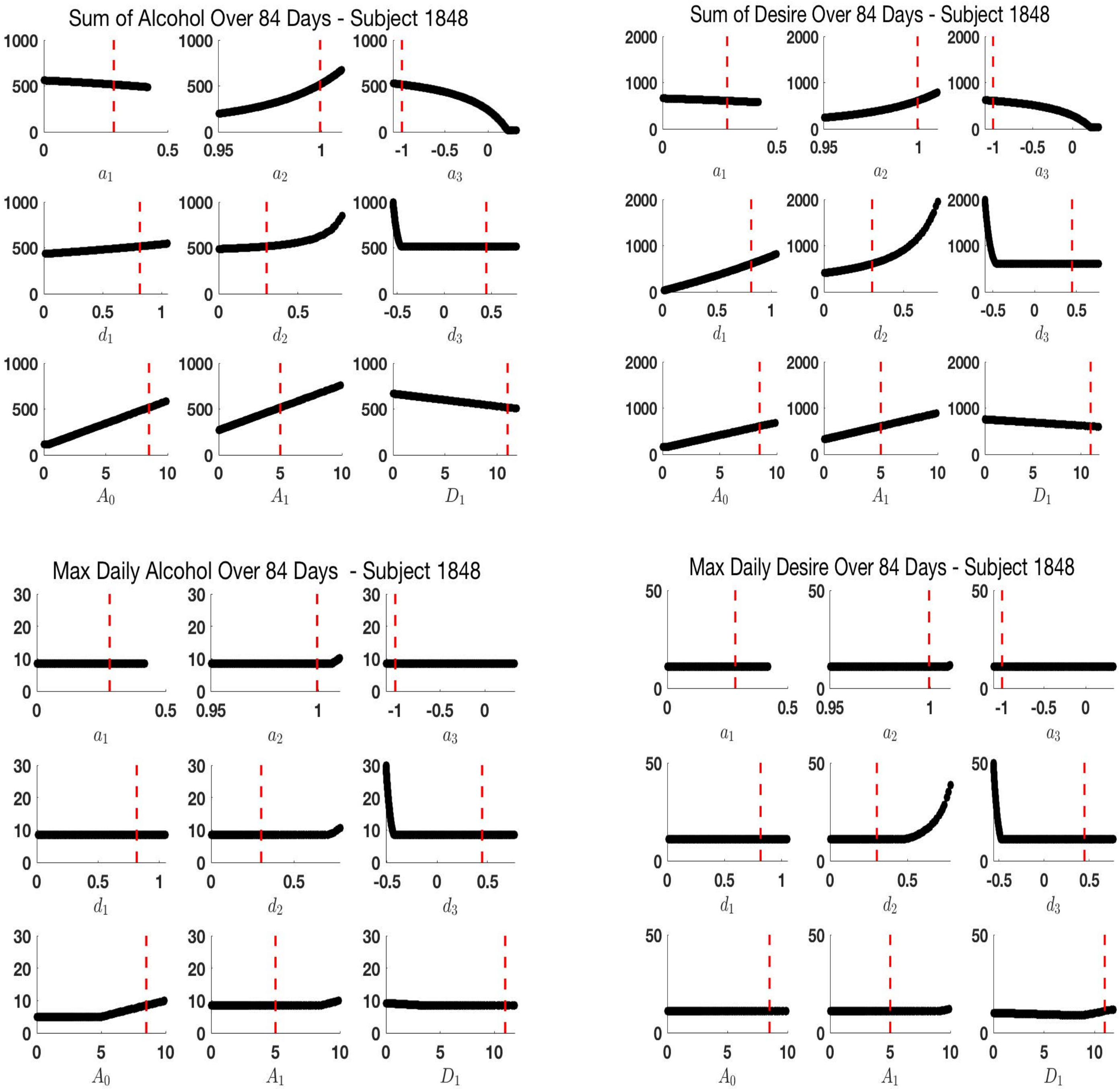

Fig S7. Relationships between varying single parameter values in the Latin Hypercube Sampling and all four output measures for 84 day observation period using parameter set for Subject 1848. The majority of plots appear to be monotonic, suggesting the PRCC is an appropriate measure to consider. When looking at the monotonic plots for this patient, we see that there are no clear mechanisms of behavior change to target given his almost steady reduction of desire and alcohol consumption. Change in any of these particular parameters will not provide a huge impact—again, indicating that intervention with this individual will not be an effective use of scarce treatment resources. This is consistent with Fig 2 that demonstrates a smooth trajectory of reduction of drinking and desire over the 84 days that this individual achieves on his own.
