# Supplementary material for: Data driven mixed effects modeling of the dual process framework of addiction among individuals with alcohol use disorder
Source: PLoS One. 2023 Aug 7;18(8):e0265168. doi: 10.1371/journal.pone.0265168 (PMC10406226; doi:10.1371/journal.pone.0265168)
Supplement: Attachment — Submitted filename: Report.pdf [file pone.0265168.s002.pdf]

Referee's report

Manuscript title: "Data driven mixed effects modeling of the dual process framework of addiction among individuals with alcohol use disorder"

The paper provides an analysis of Alcohol use disorder (AUD) using dynamical systems to calibrate a mixed effect model of the phenomenon. Both the topic and the approach are interesting. The paper is commendable as it introduces mathematical modeling and dynamical systems to an interesting field, however, a few drawbacks in terms of clarity, presentation, and discussion need to be addressed before the paper is published.

Some major points

- Although the literature review is fairly complete and adequate when it comes to AUD, it is completely inadequate when considering the use of dynamical systems in psychology. Actually, the entire section 1.3 has no references. Although dynamical systems have been used in psychology for a number of years and there are even journals devoted to this approach, the modeling approach needs to be well justified. I would start with a short paragraph describing why the use of dynamical systems is important in psychology. Then I suggest that the Authors discuss this approach and provide references to various areas of psychology that use dynamical systems. At the very least, I would mention some of the seminal contributions of van Geert in developmental psychology, and also some of the contributions of Dal Forno and collaborators in organizational psychology. Finally, there are some contributions that use dynamical systems to study addiction, see Duncan et al. (2019), among others. These contributions also need to be discussed and cited. In this way, the Authors will be able to guide the reader to understand their modeling choices.
- Information on how the subjects were recruited would be helpful. I might infer that the Authors used data discussed in a previous paper [17]. This should be clearly mentioned at the beginning of section 2.1.
- The operationalization of A_n and D_N should be clarified; I would like to know exactly how A_n and D_n were calculated from the data.
- The caption of Figures 2 is unclear. Also, in lines 174-175 the Authors claim "we use daily alcohol consumption and desire as our outcomes of interest, denoted by A_n and D_n for any given evening n, where n ranges over an 84-day". My understanding is that this means $n \in \{1, 2, \ldots, 84\}$; therefore the values on x axis are inconsistent.
- Stability of the equilibria should be analyzed and discussed.

Minor points

- Some references about data-driven modeling would be helpful.
- I find it surprising that all relationships are assumed to be linear. In several cases from the graphs in Figure 2, I see oscillating behavior. I would like to see a discussion of oscillating behavior and overshooting,

see e.g. Bickel and Rizzuto (1991, doi: 10.15288/jsa.1991.52.454), and I suggest that the Authors consider a nonlinear extension of their model in further studies.
