# Supplementary material for: Data driven mixed effects modeling of the dual process framework of addiction among individuals with alcohol use disorder
Source: PLoS One. 2023 Aug 7;18(8):e0265168. doi: 10.1371/journal.pone.0265168 (PMC10406226; doi:10.1371/journal.pone.0265168)
Supplement: Attachment — Submitted filename: Response to Reviewers.pdf [file pone.0265168.s003.pdf]

Dear Dr. Antonio Calcagni,

Thank you for your response. We have revised the manuscript according to reviewer suggestions. We provide point by point responses to each reviewer comment below. We look forward to hearing your feedback on our work.

Sincerely,

Rebecca Everett, Allison Lewis, Alexis Kuerbis, Angela Peace, Jing Li, Jon Morgenstern

---

Reviewer #1.1: Lines 131-136. The authors claim they focus on the bottom-up module of the dual-process model just to ensure successful parameterization in this “initial effort” of modeling drinking behavior. I find such an argument pretty odd. I don’t think this can be an acceptable justification to choose to focus on one part of a comprehensive cognitive process. However, mine it’s a stylistic concern. Indeed, modeling (only) a bottom-up process is, in general, a challenging task which already justifies the effort. Maybe the authors might be interested in replacing the justification for focusing on just the bottom-up process, with an explanation of why it is important to take into account bottom-up processes of addictive behavior, by providing a more substantiated justification for the modeling choice.

Paragraphs 2.3 and 2.4 should be definitely improved from this perspective. As it is now, the manuscript provides a rough introduction to what bottom-up and top-down processes in addiction are, and an apparently pointless explanation on why one of the processes has been dropped. The arguments about the eventual extensions of the model, as well as the necessity to include also the top-down part, can be directly moved to the discussion section.

Author response: We thank the reviewer for their thoughtful comment on the theoretical basis of our model building process. We agree that our approach is reductionistic, and, in fact, we started with all of the components of the dual process model outlined in Figure 1. It quickly became clear that beginning with all components at once created too much noise in our model building process—it was difficult to identify which components were strong and a good fit versus those that were detracting from model fit. Thus, we started with one component, with the intention of adding additional components in the future.

To address this issue in the paper, taking the guidance of the reviewer, we have added justification for why the bottom-up processes are important as a focus on their own, highlighting the importance of craving as a new criterion of AUD and a strong predictor of sustained hazardous drinking. Additionally, we have moved the mention of our future aims to the discussion section.

Reviewer #1.2: Line 143. Is it possible to know why all observations were bounded at two standard deviations?

Author response: Observations were bounded at two standard deviations, or winsorized, in order to reduce the impact of extreme outliers. In this case, it also made responses more clinically relevant. When participants were asked the number of drinks they had last night, responses ranged from 0 to 101. 98% of the responses were 17 and below. While it is *possible* that a person could drink that in a day, in the context of extremely high alcohol tolerance, it is unlikely (especially given extreme physiological dependence as an exclusion criterion). Therefore, extreme outliers are likely typos (respondents were using a touch pad on a telephone to answer questions). Out of 3108 observations, 44 were winsorized to 17. We have added this fuller explanation to the paper (see Section 2.3).

Reviewer #1.3: Section 3.1. Is there any particular reason why you decided to use such a flow of information presentation? I'm referring to the way in which you described the model and parameters. In general, cognitive modeling papers present the following structure when describing a model (with some exception): (1) presentation of non-parameter elements (e.g. A_n in your case), such as predictors or observed data and their structure and meaning; (2) the equation embedding observed data and parameters; (3) description of the parameters. Of course, this is not mandatory at all, but I find the description logic here really odd. In general, you presented, literally, chunks, or pieces, of the final model (e.g. a_2 * A_{n-1}) with a description of the parameters, first. After dozens of lines, the whole model (e.g. equations) is provided. I think that it is more readable to describe observed variables first, and then providing a description of the parameters after presenting the whole model. But it's your choice.

Author response: Following these suggestions, we reorganized the model description and added several clarifications. We first introduce the state variables and present the model at the beginning of Section 3.1. We include a parameter table summarizing all the model parameters. Then the following subsections give details on the model parameters.

Reviewer #1.4: Section 4.2. Here, more details about the Bayesian parameter estimation setup and procedure have to be provided. First, you should clarify whether and how you specified a likelihood function, as well as the distributional assumption related (e.g. the distributional model of the dependent variables set, and the assumption related to the (in)dependency of the observations). Second, you should show and justify the prior distributions over the free parameters, including the prior over the variance/covariance matrix. Such details can be moved to a "Model Specification" section if needed.

Author response: We thank the reviewer for pointing out our lack of clarity in this area. Section 4.2 has been expanded to include a discussion of the specific likelihood function and prior distributions employed for this particular scenario.

Reviewer #1.5: Lines 330-338. Authors show that the model is not identifiable for some individuals by means of some thresholded scoring procedure, and also provide values for fixed parameters based on some statistics computed on the whole sample. However, since the manuscript is presented as a work with empirical implications, I would expect some clarification about how a researcher, or a clinician, should use the proposed tool in practice. For instance, I

would like to know which pipeline authors advise when dealing with empirical data, to deal with individual-specific non-identifiability, or fixed parameters based on the current sample.

Author response: In Section 4.1, we added text clarifying that the collinearity method is highly dependent upon the initial parameter guess; since all four of the unidentifiable patients had outlier parameter guesses, we recommend a possible method to use in clinical practice for choosing an alternate starting point which could result in acceptable collinearity scores. Since this is a proof-of-concept analysis, and in this particular investigation three out of the four collinearity scores are close to the arbitrary threshold value, we proceed with estimation on the four-parameter subset.

Reviewer #1.6: Lines 478-493. I think the interpretation of parameter estimates should be provided after mixed-effects model results, to improve readability. Indeed, paragraph 5.1 ends up abruptly, and parameter estimates are not discussed enough.

Author response: Following the suggestion, we moved these lines from Section 6 (Discussion) to Section 5.1 (Mixed effects model results).

Reviewer #1.7: Lines 495-. I found the sensitivity analysis paragraphs in the discussion particularly meaningful and interesting. But I can't see the point in having such sections, as they are conceived right now, in the separate discussion. They contain important insights about how to interpret model results, and how to use model results. Is there any particular reason (both conceptual or stylistic) why (at least some of) the contents of such paragraphs should not be included in the main sections describing the analysis? As it is now, the reading results in a pretty odd and inconsistence experience, in which you read a fragment of the story (e.g. a piece of model fitting results, a piece of sensitivity analysis results), and you have to wait for some paragraph before reading the other part of the story. I'm not saying that discussions should not contain arguments related to the analysis performed in the paper, but I'm saying that there are some contents (such are the direct interpretation of parameter estimation results) which should be embedded in the main sections.

Author response: Our choice to include these sensitivity analysis paragraphs in the Discussion Section were based on the fact that we are aiming for an audience of both applied mathematicians and social scientists who structure papers differently. Following the typical paper structure used by social scientists, we decided to include only raw results in the Results section and leave interpretation of results for the Discussion section. We appreciate that this might seem counterintuitive to some readers, but we decided upon this organization in order to reach a wide audience.

Reviewer #1.8: Please follow the standard Bayesian pipeline by presenting all the metrics to assess chains convergence, such as R-hat, Bulk- and Tail- Effective Sample Size, Autocorrelation functions.

Author response: We thank the reviewer for this suggestion. We now include a section in the Supplementary Material that includes several diagnostic criteria for chain convergence,

including MC errors, Geweke p-values, and autocorrelation results, as well as a visualization of the chains and autocorrelation plots for the four population level parameters.

Reviewer #1.9: Figure's quality is very bad. Please improved.

Author response: We apologize and have improved the quality of the figures.

Reviewer #2.1: Although the literature review is fairly complete and adequate when it comes to AUD, it is completely inadequate when considering the use of dynamical systems in psychology. Actually, the entire section 1.3 has no references. Although dynamical systems have been used in psychology for a number of years and there are even journals devoted to this approach, the modeling approach needs to be well justified. I would start with a short paragraph describing why the use of dynamical systems is important in psychology.

Then I suggest that the Authors discuss this approach and provide references to various areas of psychology that use dynamical systems. At the very least, I would mention some of the seminal contributions of van Geert in developmental psychology, and also some of the contributions of Dal Forno and collaborators in organizational psychology. Finally, there are some contributions that use dynamical systems to study addiction, see Duncan et al. (2019), among others. These contributions also need to be discussed and cited. In this way, the Authors will be able to guide the reader to understand their modeling choices.

Author response: We thank the reviewer for their thoughtful comments about dynamical systems modeling within psychology as a whole, as well as pointing us to seminal and recently published work, especially in addiction. Our omissions related to the history of dynamic systems modeling in psychology was not meant to be dismissive. Its purpose was parsimony, given the extensive length of the paper thus far. As suggested by the reviewer, we have added paragraphs in Section 1.3 on previous work and its importance to psychology and addiction specifically.

Reviewer #2.2: Information on how the subjects were recruited would be helpful. I might infer that the Authors used data discussed in a previous paper [17]. This should be clearly mentioned at the beginning of section 2.1.

Author response: In the beginning paragraph of section 2, just before section 2.1, we previously stated directly that these data are from a previous study, discussed in a previous paper. As recommended, we have now moved this to the beginning of section 2.1, as well as added recruitment procedures to section 2.1. We hope this clarifies the origin of the data and recruitment procedures.

Reviewer #2.3: The operationalization of A n and D N should be clarified; I would like to know exactly how A n and D n were calculated from the data.

Author response: Throughout the manuscript, we clarified the differences between the data and model variables. In section 3, we now state that An and Dn are the state variables in the model - that is, they are not calculated from the data, but from the model (equations 1a and 1b).

Reviewer #2.4: The caption of Figures 2 is unclear. Also, in lines 174-175 the Authors claim “we use daily alcohol consumption and desire as our outcomes of interest, denoted by A n and D n for any given evening n, where n ranges over an 84-day”. My understanding is that this means n $\in \{1, 2, . . . , 84\}$; therefore the values on x axis are inconsistent.

Author response: We have changed the axis labels in Figure 2 (now Figure 1) to clarify what is plotted in each figure, and to no longer refer to the variables An and Dn. Time is not on the horizontal axis, which is why the horizontal axis does not range from 1 to 84. We hope that these changes along with the clarifying differences between the data and model variables throughout the manuscript make this Figure more clear.

Reviewer #2.5: Stability of the equilibria should be analyzed and discussed.

Author response: We appreciate the reviewer’s suggestion about including stability of equilibria. However, given the complexity of the model as a 2nd order discrete dynamical system, determining the stability of the equilibria is a non-trivial task and beyond the scope of this paper. We leave this as future work.

Reviewer #2.6: Some references about data-driven modeling would be helpful.

Author response: We have incorporated some references in Section 2.4.

Reviewer #2.7: I find it surprising that all relationships are assumed to be linear. In several cases from the graphs in Figure 2, I see oscillating behavior. I would like to see a discussion of oscillating behavior and overshooting, see e.g. Bickel and Rizzuto (1991, doi: 10.15288/jsa.1991.52.454), and I suggest that the Authors consider a nonlinear extension of their model in further studies.

Author response: Thank you for these suggestions. During the model development we had thorough discussions on including nonlinear relationships but the empirical data in Figure 2 (now Figure 1) motivated us to assume linear relationships. Firstly, we clarified the axes in Figure 2 (now Figure 1). While some of the data points suggest nonlinear relationships, the red points were calculated from limited data, as described in the caption; note that the black dots in Figure 2 (now Figure 1) are determined from an average of 120 data points but the red dots are calculated from fewer than 10 data points. Therefore, the majority of the empirical data suggests linear relationships rather than nonlinear ones. Additionally, if we were to assume non-linear relationships we would have to parameterize these relationships based on limited amounts of data. We decided to build this initial model using linear relationships and mentioned investigations of nonlinear ones as future work.

It is also important to note that, while we did assume linear relationships in the model (equations 1a and 1b), the model output did predict oscillatory behaviors for some individuals - see grey model predictions in Figure 2.
